# Retraction of: P-1835. Dual burden of infections: seroprevalence of acute viral hepatitis among dengue patients in northern India

**DOI:** 10.1093/ofid/ofag305

**Published:** 2026-06-11

This is a retraction of Monirujjaman Biswas, P-1835. Dual burden of infections: seroprevalence of acute viral hepatitis among dengue patients in northern India, *Open Forum Infectious Diseases*, Volume 13, Issue Supplement_1, January 2026, ofaf695.2004, https://doi.org/10.1093/ofid/ofaf695.2004.

The journal is retracting this IDWeek abstract because there is significant overlap between it and a previously published abstract without attribution: A retrospective analysis on seroprevalence of acute viral hepatitis observed among dengue patients attending a tertiary care centre in central India, Jain et al. (https://doi.org/10.1016/j.ijmmb.2024.100572). Additionally, the authorship of the publication cannot be verified.

